# Pre-clinical medical student cardiac point-of-care ultrasound curriculum based on the American Society of Echocardiography recommendations: a pilot and feasibility study

**DOI:** 10.1186/s40814-021-00910-3

**Published:** 2021-09-14

**Authors:** Satoshi Jujo, Jannet J. Lee-Jayaram, Brandan I. Sakka, Atsushi Nakahira, Akihisa Kataoka, Masaki Izumo, Kenya Kusunose, Natsinee Athinartrattanapong, Sayaka Oikawa, Benjamin W. Berg

**Affiliations:** 1 SimTiki Simulation Center, John A. Burns School of Medicine, University of Hawaii at Manoa, 651 Ilalo St, MEB 212, Honolulu, HI 96813 USA; 2 Department of Anesthesiology, Kameda General Hospital, Chiba, Japan; 3 Department of Critical Care Medicine, Nara Prefecture General Medical Center, Nara, Japan; 4 Division of Cardiology, Department of Internal Medicine, Teikyo University, Tokyo, Japan; 5 Division of Cardiology, Department of Internal Medicine, St. Marianna University School of Medicine, Kanagawa, Japan; 6 Department of Cardiovascular Medicine, Tokushima University Hospital, Tokushima, Japan; 7 Department of Emergency Medicine, Ramathibodi Hospital, Mahidol University, Bangkok, Thailand; 8 Center for Medical Education and Career Development, Fukushima Medical University, Fukushima, Japan

**Keywords:** Medical student, Medical education, Point-of-care ultrasound, POCUS, Handheld ultrasound, Echocardiography, Curriculum development

## Abstract

**Background:**

Cardiac point-of-care ultrasound (POCUS) training has been integrated into medical school curricula. However, there is no standardized cardiac POCUS training method for medical students. To address this issue, the American Society of Echocardiography (ASE) proposed a framework for medical student cardiac POCUS training. The objective of this pilot study was to develop a medical student cardiac POCUS curriculum with test scoring systems and test the curriculum feasibility for a future definitive study.

**Methods:**

Based on the ASE-recommended framework, we developed a cardiac POCUS curriculum consisting of a pre-training online module and hands-on training with a hand-held ultrasound (Butterfly iQ, Butterfly Network Inc., Guilford, CT, USA). The curriculum learning effects were assessed with a 10-point maximum skill test and a 40-point maximum knowledge test at pre-, immediate post-, and 8-week post-training. To determine the curriculum feasibility, we planned to recruit 6 pre-clinical medical students. We semi-quantitatively evaluated the curriculum feasibility in terms of recruitment rate, follow-up rate 8 weeks after training, instructional design of the curriculum, the effect size (ES) of the test score improvements, and participant satisfaction. To gather validity evidence of the skill test, interrater and test-retest reliability of 3 blinded raters were assessed.

**Results:**

Six pre-clinical medical students participated in the curriculum. The recruitment rate was 100% (6/6 students) and the follow-up rate 8 weeks after training was 100% (6/6). ESs of skill and knowledge test score differences between pre- and immediate post-, and between pre- and 8-week post-training were large. The students reported high satisfaction with the curriculum. Both interrater and test-retest reliability of the skill test were excellent.

**Conclusions:**

This pilot study confirmed the curriculum design as feasible with instructional design modifications including the hands-on training group size, content of the cardiac POCUS lecture, hands-on teaching instructions, and hand-held ultrasound usage. Based on the pilot study findings, we plan to conduct the definitive study with the primary outcome of long-term skill retention 8 weeks after initial training. The definitive study has been registered in ClinicalTrials.gov (Identifier: NCT04083924).

**Supplementary Information:**

The online version contains supplementary material available at 10.1186/s40814-021-00910-3.

## Key messages regarding feasibility

1. **What uncertainties existed regarding the feasibility?**

Based on the American Society of Echocardiography (ASE) recommendations, we developed a medical student cardiac point-of-care ultrasound (POCUS) curriculum and assessment tools for the skill and knowledge learning effects of the curriculum to determine the short- and long-term retention. The feasibility of the cardiac POCUS curriculum and the assessment tool validity were uncertain.

2) **What are the key feasibility findings?**

This pilot study confirmed the feasibility of the cardiac POCUS curriculum without recruitment and attrition concerns. The curriculum resulted in large effect sizes of skill and knowledge learning effects. The assessment tools gathered validity evidence from Messick’s framework including content, response process, and internal structure for future use.

3) **What are the implications of the feasibility findings for the design of the main study?**

This formal pilot study provided insight into the medical student cardiac POCUS curriculum and resulted in instructional design changes to enhance the likelihood of success of the definitive study. Based on the pilot study findings, we plan to conduct the definitive study with the primary outcome of long-term skill retention 8 weeks after initial training.

## Introduction

As technology advances, cardiac point-of-care ultrasound (POCUS) with hand-held ultrasound devices (HHU) is being used increasingly in clinical practice by multiple specialties including cardiology, emergency medicine, critical care medicine, anesthesiology, and family medicine [1–5]. In 2019, HHU that operate with smartphones or tablets became commercially available (https://www.butterflynetwork.com/). The image quality is high enough for cardiac assessment and the costs are touted as affordable to even medical students [6]. There is a foreseeable future where medical student stethoscopes could be replaced with HHU or “ultrasound stethoscopes” for learning cardiovascular clinical examinations [7].

With growing student interest in cardiac ultrasound examination [8], multiple studies have demonstrated feasibility and benefits of integrating cardiac POCUS into the undergraduate medical education (UME) curriculum [9–14]. A comprehensive review article on cardiac POCUS in medical school education described that 12 medical schools in the United States, Canada, Norway, United Kingdom, Israel, Poland, and China have incorporated cardiac POCUS curricula [15]. However, instructional design for teaching cardiac POCUS to medical students is highly variable, without validated or standardized methodology regarding training duration, teaching methods, and competency evaluation [15].

In an effort to address this issue, the American Society of Echocardiography (ASE) proposed a framework for medical student cardiac POCUS teaching for UME program directors wishing to institute POCUS curricula at their schools (https://aselearninghub.org/) [15]. The ASE framework includes a pre-training didactic education with e-learning, hands-on training, and a competency evaluation. The goals are to enhance cardiac physical examination skills and augment learning of normal anatomy, rather than learning advanced pathology. At this time, the effectiveness of the ASE-recommended framework has not been evaluated.

Therefore, the primary objective of this pilot study was to develop a medical student cardiac POCUS curriculum based on the ASE recommendations and evaluate the curriculum feasibility for a definitive study. A secondary objective was to assess the curriculum learning effects in order to identify a single primary outcome for a definitive study.

## Methods

### Design

The study was approved by the University of Hawaii Human Studies Program and was conducted between July 2019 and September 2019 (Protocol number: 2019-00265).

This pilot study was comprised of 4 elements:

(i) Development of a cardiac POCUS curriculum for pre-clinical medical students,
(ii) Evaluation of curriculum feasibility and further modifications needed for a definitive study,
(iii) Development of an assessment tool to evaluate curriculum learning effects and gathering validity evidence of the tool, and
(iv) Assessment of the curriculum’s multiple learning effect outcomes in order to determine a single primary outcome for the definitive study. We conducted this pilot study based on guidelines for reporting pilot and feasibility studies [16–18] with the Consolidated Standards of Reporting Trials (CONSORT) extension to pilot and feasibility trials checklist (Additional File 1).

### Participants and setting

We planned to recruit 6 pre-clinical medical students to determine the curriculum feasibility. Eligible participants were first- or second-year medical students who had completed a 12-week pre-clinical cardiovascular and pulmonary core curriculum at the University of Hawaii, John A. Burns School of Medicine (JABSOM) in the United States. We used e-mail and public postings at JABSOM for recruiting participants. One of the investigators (BS) was a medical student at JABSOM and approached his colleagues by word of mouth to invite them to participate.

### Cardiac POCUS curriculum development

**Fig. 1:**
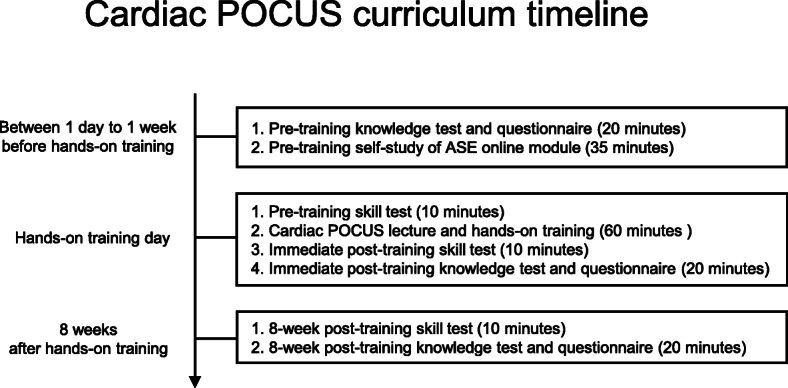
Cardiac point-of-care ultrasound curriculum timeline. *POCUS*, point-of-care ultrasound; *ASE*, American Society of Echocardiography

We developed a basic cardiac POCUS curriculum for pre-clinical medical students based on the ASE-recommended framework that encourages using a blended learning system with a flipped classroom model [15, 19]. The notions of curriculum design, teaching methods, and competency assessments were underpinned by educational principles for effective learning and skill retention [20], which include *concurrent feedback*, *deliberate practice*, *mastery learning*, and *range of difficulty*. Student goals for this curriculum were to independently obtain basic cardiac POCUS views in a healthy volunteer and identify the normal anatomic structures seen in the POCUS views. Curriculum developers were educational methods and echocardiography subject matter experts; a cardiovascular anesthesiologist specializing in perioperative transesophageal echocardiography and critical care echocardiography (SJ), a pediatric emergency physician and simulation-based education specialist (JL), a cardiac surgeon (AN), 3 cardiologists specializing in echocardiography and echocardiography education in their university hospitals (AK, MI, KK), a Fellow of the American Society of Echocardiography (FASE) certified echocardiographer (KK), and an adult intensivist and simulation-based education specialist (BB).The curriculum timeline is shown in Fig. 1. The cardiac POCUS curriculum included a pre-training self-study of the ASE cardiac POCUS online module and a healthy male volunteer 5-view cardiac POCUS hands-on training session (cardiac POCUS lecture and hands-on training). The students used an HHU probe (Butterfly iQ; Butterfly Network, Inc., Guilford, CT, USA) with a 9.7-inch tablet display during the curriculum. Student skill and knowledge were assessed pre-training and immediately post-training with a 10-point maximum skill test and a 40-point maximum knowledge test. Additionally, we evaluated student skill and knowledge retention at 8 weeks post-training with the identical tests. The 8 weeks post-training timeframe for retention assessment was chosen based on previous studies that demonstrated clinical skill temporal degradation in medical students 6-12 weeks after training [21] and described the typical time course of basic POCUS skill degradation in medical students to be within 8 weeks [22].

#### ASE cardiac POCUS online module for medical students

The ASE POCUS task force published a free cardiac POCUS online module for medical students in 2018 (https://aselearninghub.org/). We utilized the ASE online module entitled “Cardiovascular Point-of-Care Imaging for the Medical Student and Novice User” as the pre-training didactic. The complete ASE online module is comprised of 8 sub-modules: *Introduction*, *Basic Anatomy Correlating to Cardiac POCUS Views (Module A)*, *Complete Cardiac POCUS Scan (Module B)*, *Integrated Cardiac Point-of-Care and Physical Exam (Module C)*, *Pathology-I (Module D)*, *Pathology-II (Module D)*, *Teaching the Teacher (Module E)*, *and Standards and Testing (Module F)*. Our pre-training self-study curriculum included the first 4 ASE modules on normal anatomy and physiology (*Introduction*, *Module A*, *B*, *and C*), which were matched to the learner level of pre-clinical medical students without extensive prior knowledge of cardiac pathology. The 4 modules were designed to be completed in approximately 35 min. Students independently reviewed the online modules between 1 day to 1 week before hands-on training. Duration of student online review was not tracked.

#### cardiac POCUS views selection

We selected 5 cardiac POCUS views to teach during the hands-on training session; parasternal long-axis (PLAX), papillary muscle level of parasternal short-axis (PSAX), apical 4-chamber (A4C), subcostal 4-chamber (S4C), and subcostal inferior vena cava (SIVC) view. The ASE-recommended framework does not mention specific cardiac POCUS views to teach [15], while the ASE online module provides 10 cardiac ultrasound views. However, teaching all 10 views in the first 4 ASE modules was inappropriate for initial medical student training [10]. Selection of the 5 views was based on review of 2 guidelines and 3 studies: the World Interactive Network Focused on Critical UltraSound (WINFOCUS) recommendations [4], the European Association of Cardiovascular Imaging (EACVI) consensus for focused cardiac ultrasound (FoCUS) [23], a Focused Echocardiography Entry Level (FEEL) training study [24], a medical student FoCUS training study [14], and an initial transthoracic echocardiography (TTE) training study for anesthesiologists [25]. The WINFOCUS recommendations and the EACVI consensus selected the same 5 views as an ideal standard or basic for cardiac POCUS examination, and the 3 studies utilized the same 5 views for hands-on training. The ASE recently published cardiac POCUS and critical care echocardiography (CCE) training recommendations that also supported our 5 views selection for initial cardiac POCUS learning tasks [3].

#### Cardiac POCUS hands-on training session

The cardiac POCUS hands-on training session was comprised of a pre-training skill test (10 min), a cardiac POCUS lecture and hands-on training (60 min), and an immediate post-training skill test (10 min). All hands-on training sessions were conducted by a single instructor (SJ). Immediately prior to the start of the hands-on training session, we assessed student baseline 5-view cardiac POCUS image acquisition skill (Pre-training skill test). After the baseline skill assessment, the instructor guided a 30-min interactive lecture using PowerPoint slides and a model heart (Cardiac POCUS lecture). The lecture included didactic content regarding basic 5 cardiac POCUS views and acoustic windows using the ASE online module slides and pre-recorded video instructions. The video instructions, which we created, demonstrated the 5-view image acquisition on the healthy volunteer. The lecture content is summarized in Additional File 2. Image acquisition instructional design in the video instructions was created with reference to an imaging protocol in the ASE comprehensive TTE guidelines [26] and a point-of-care ultrasound textbook [27]. The main instruction points for the 5-view image acquisition are summarized in Additional File 3. Following the cardiac POCUS lecture, students engaged in a 30-min supervised hands-on training of the 5-view image acquisition on a thin, healthy volunteer (Cardiac POCUS hands-on training). The instructor (SJ) assumed the role of the healthy volunteer during the hands-on training and, at the same time, provided concurrent, verbal, and tactile feedback. In the hands-on training, students practiced the image acquisition repeatedly until they were able to obtain each image clearly. In order to determine a feasible group size for the hands-on training session, we trialed 3 different group sizes: one 3-student group, one 2-student group, and one 1-student group. Immediately after and 8 weeks after the hands-on training session, we reassessed student cardiac POCUS skill with the same skill test (Immediate post-training skill test and 8-week post-training skill test, respectively).

### Skill test scoring system

#### Skill test

We assessed student cardiac POCUS skill at pre-training, immediate post-training, and 8-week post-training using a skill test scoring system that we developed. During the skill test, students independently demonstrated the 5 cardiac POCUS views in a fixed sequence (PLAX, PSAX, A4C, S4C, and SIVC) on the same single healthy volunteer without guidance. Students were limited to 2 min to obtain each view, for a total of 10 min for 5 views. During the 2-min image acquisition interval, students self-identified their “best” view and pressed a recording button to acquire a 5-second video clip. Students were allowed to record a maximum of 2 video clips for each view. If they recorded 2 video clips, they chose the single best video clip without review as the recording to be evaluated for this study. We utilized the Butterfly iQ application predefined cardiac ultrasound preset for gain and other ultrasound imaging parameters [28]. The healthy volunteer took the left decubitus position for PLAX, PSAX, and A4C, and the supine position with bent knees for S4C and SIVC, and controlled his respiratory rate at 6 per minute and held his breath for 5 seconds when the video clip recording started. Students, therefore, focused only on probe manipulation to obtain each view and did not adjust ultrasound imaging parameters, the healthy volunteer’s position, or breathing cycle during the skill test.

#### 10-point maximum skill test scoring system

**Fig. 2:**
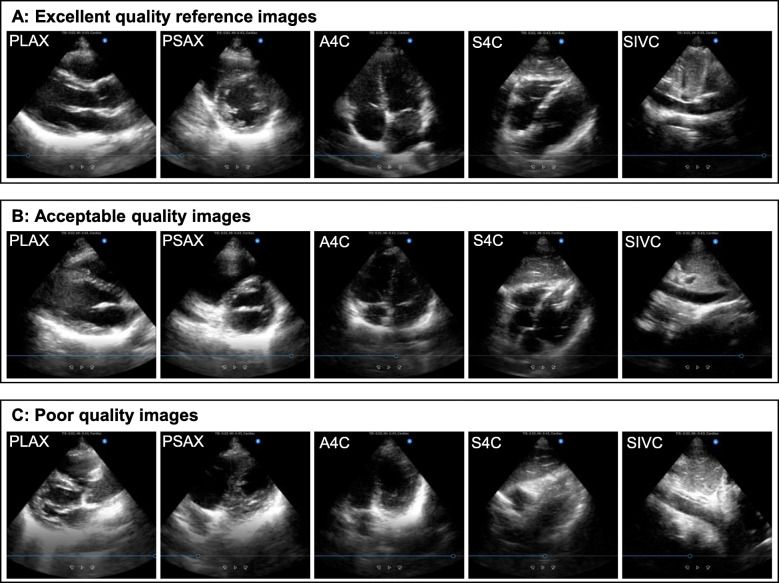
Excellent quality reference (**A**) and examples of acceptable (**B**) and poor quality (**C**) images of 5 cardiac POCUS views. Legend: Excellent quality reference images (**A**) refer to 5 cardiac POCUS views obtained by the cardiologist (MI) on the healthy volunteer (SJ) used for all skill tests. Examples of acceptable (**B**) and poor quality (**C**) images refer to the 5 views obtained by medical students on the volunteer. The excellent quality reference image (**A**) of the PLAX demonstrates all 7 structures (LA, LV, LVOT, RV, AV, MV, and IVS); the PSAX demonstrates all 4 structures (round LV, RV, papillary muscles, and IVS); the A4C demonstrates all 8 structures (LA, LV, RA, RV, MV, TV, IAS, and IVS); the S4C demonstrates all 7 structures (LA, LV, RA, RV, IAS, IVS, and liver); and the SIVC visualizes the IVC in a longitudinal fashion, the clear connection of IVC to RA, and the IVC diameter ≥ 1.0 cm at 2 cm from RA-IVC junction. The acceptable quality image (**B**) of the PLAX does not visualize AV; the PSAX is the mitral level of parasternal long-axis view, not the papillary muscle level; the A4C demonstrates a severely foreshortened LV; the S4C misses the LA and adds the aortic outflow (5-chamber view); and the SIVC does not visualize a clear connection of IVC to RA. The poor quality image (**C**) of the PLAX does not visualize the AV and the LV is severely foreshortened; the PSAX does not demonstrate a round LV and misses the papillary muscles; the A4C demonstrates a severely foreshortened LV and misses the LA; the S4C misses the RV and does not visualize the LA and the LV well; the SIVC demonstrates the descending aorta instead of the IVC. *POCUS*, point-of-care ultrasound; *PLAX*, parasternal long-axis view; *PSAX*, papillary muscle level of parasternal short-axis view; *A4C*, apical 4-chamber view; *S4C*, subcostal 4-chamber view; *SIVC*, subcostal inferior vena cava view; *LA*, left atrium; *LV*, left ventricle; *LVOT*, left ventricle outflow tract; *RV*, right ventricle; *AV*, aortic valve; *MV*, mitral valve; *IVS*, interventricular septum; *RA*, right atrium; *TV*, tricuspid valve; *IAS*, interatrial septum; *IVC*, inferior vena cava

We developed a 10-point maximum scoring system by modifying an existing assessment tool for TTE views [25]. The scoring system was designed to assess 5-view image quality for the purpose of rapid bedside cardiac assessment, not for a formal diagnostic comprehensive echocardiography examination. Scoring system development details are shown in Additional File 4. The 10-point maximum skill test scoring system rated the 5 views; each view was assessed as excellent (2 points), acceptable (1 point), or poor (0 point) for cardiac POCUS use (Table 1). Excellent quality reference images of the 5 views obtained by the cardiologist (MI) on the healthy volunteer (SJ) are shown in Fig. 2A. Examples of acceptable and poor quality images are shown in Fig. 2 B and C, respectively. After de-identifying the skill test video clips including removal of pre-, immediate post-, and 8-week post-training status, we arranged the clips in a randomized order using the Microsoft Excel random number table for blinded assessment. Three independent blinded raters scored image quality using the 10-point maximum skill test scoring system, and the average score from the 3 raters was utilized as a representative score. Interrater reliability of the skill test scoring system was assessed with intraclass correlation coefficient (ICC) using 10-point maximum skill test scores (pre-, immediate post-, and 8-week post-training) and 2-point maximum scores from each of 5 views (PLAX, PSAX, A4C, S4C, and SIVC) from all students. Test-retest reliability of the 3 raters was assessed with ICC by re-assessing the skill test video clips after de-identification. The 3 raters were echocardiography experts who developed the skill test scoring system and were blinded to student identity and timing of the assessment. If interrater reliability and/or test-retest reliability were moderate or poor (ICC < 0.75), we planned to re-evaluate the scoring system or perform rater training.

**Table 1:** 10-point maximum skill test scoring system

5 cardiac POCUS views	Points	Image quality criteria	
Parasternal long-axis view	2	Excellent:	All 7 chambers and anatomical structures (LA, LV, LVOT, RV, AV, MV, and IVS) visualized or similar to the excellent quality reference^a^.
	1	Acceptable:	One chamber (LA, LV, or RV) severely foreshortened or one anatomical structure (LVOT, AV, MV, or IVS) not visualized well.
	0	Poor:	Any two of chambers or structures (LA, LV, LVOT, RV, AV, MV, and IVS) severely foreshortened/not visualized well, the left and right sides of the image are flipped, raters do not recognize the view as a parasternal long-axis view, or no image obtained.
Parasternal short-axis view (papillary muscle level)	2	Excellent:	All 4 chambers and anatomical structures (round LV, RV, papillary muscles, and IVS) visualized or similar to the excellent quality reference^a^.
	1	Acceptable:	One chamber or anatomical structure (round LV, RV, papillary muscles, or IVS) not visualized well, oval LV, significant lateral wall drop out of LV compared to the excellent quality reference^a^, or mitral level of parasternal short-axis view.
	0	Poor:	Any two of chambers or anatomical structures (round LV, RV, papillary muscles, and IVS) not visualized well, apical level or aortic valve level of parasternal short-axis view, the left and right sides of the image are flipped, raters do not recognize the view as a parasternal short-axis view, or no image obtained.
Apical 4-chamber view	2	Excellent:	All 8 chambers and anatomical structures (LA, LV, RA, RV, MV, TV, IAS, and IVS) visualized or similar to the excellent quality reference^a^.
	1	Acceptable:	One chamber (LA, LV, RA, or RV) severely foreshortened, one anatomical structure (MV, TV, IAS, or IVS) not visualized well, aortic outflow added (5-chamber view), or significant lateral wall drop out of LV compared to the excellent quality reference^a^.
	0	Poor:	Any two of chambers or anatomical structures (LA, LV, RA, RV, MV, TV, IAS, and IVS) not visualized well, the left and right sides of the image are flipped, raters do not recognize the view as a apical 4-chamber view, or no image obtained.
Subcostal 4-chamber view	2	Excellent:	All 7 chambers and anatomical structures (LA, LV, RA, RV, IAS, IVS, and liver) visualized or similar to the excellent quality reference^a^.The left and right side flipped image does not affect the subcostal 4-chamber view scoring.
	1	Acceptable:	One chamber or anatomical structure (LA, LV, RA, RV, IAS, IVS, or liver) severely foreshortened/not visualized well or aortic outflow added (5-chamber view).
	0	Poor:	Any two of chambers or anatomical structures (LA, LV, RA, RV, IAS, IVS, and liver) not visualized well, raters do not recognize the view as a subcostal 4-chamber view, or no image obtained.
Subcostal IVC view	2	Excellent:	IVC visualized in a longitudinal fashion, connection of IVC to RA visualized clearly, and IVC diameter ≥ 1.0 cm at 2 cm from RA-IVC junction, or similar to the excellent quality reference^a^. The left and right side flipped image does not affect the subcostal IVC view scoring.
	1	Acceptable:	IVC diameter ≥ 1.0 cm at 2 cm from RA-IVC junction, but no clear connection of IVC to RA, or IVC not visualized in a longitudinal fashion.
	0	Poor:	IVC diameter < 1.0 cm at 2 cm from RA-IVC junction, descending aorta imaged instead of IVC, raters do not recognize the view as a subcostal IVC view, or no image obtained.

*POCUS*, point-of-care ultrasound; *LA*, left atrium; *LV*, left ventricle; *LVOT*, left ventricle outflow tract; *RV*, right ventricle; *AV*, aortic valve; *MV*, mitral valve; *IVS*, interventricular septum; *RA*, right atrium; *TV*, tricuspid valve; *IAS*, interatrial septum; *IVC*, inferior vena cava

^a^Excellent quality reference refers to an image obtained by the cardiologist (MI) on the healthy volunteer used for all skill tests (Fig. 2)

NOTE: The 2-point maximum scores for each of the 5 cardiac POCUS views are added for the 10-point maximum skill test score

### Knowledge test scoring system

We assessed student cardiac POCUS knowledge at pre-training, immediate post-training, and 8-week post-training using an identical knowledge test on Google Forms. The knowledge test consisted of 40 multiple-choice questions regarding the identification of normal anatomic structures seen in the 5 cardiac POCUS views. The 40-point maximum knowledge test scoring system is shown in Additional File 5. Students independently completed the pre-training knowledge test prior to reviewing the ASE online module. Students independently completed the immediate post-training knowledge test after the hands-on training session and the 8-week post-training knowledge test after the 8-week post-training skill test (Fig. 1).

### Butterfly iQ hand-held ultrasound

The Butterfly iQ HHU measures 185 × 56 × 35 mm and weighs 313 g, which is connected by wire to a smartphone or tablet with the Butterfly iQ application. The application features 19 selectable presets of optimal imaging parameter values including abdomen, cardiac, lung, and vascular options. The application automatically operates in accordance with a selected preset. The application has touch-screen controls including those for image recording and freezing, and depth and gain adjustment. The battery is designed for run time of over 2 h in B-mode. Ultrasound image recordings can be saved in the application, uploaded to an secure commercial cloud, and downloaded to a computer after data encryption (https://www.butterflynetwork.com/) [28].

### Curriculum learning effect outcomes

We measured 7 curriculum learning effect outcomes: [i]–[vii].

#### Skill test score

[i] skill test score difference between pre-training and immediate post-training and [ii] the difference between pre-training and 8-week post-training.

#### Knowledge test score

[iii] knowledge test score difference between pre-training and immediate post-training and [iv] the difference between pre-training and 8-week post-training.

#### Post-training questionnaire

We used 5-point Likert scales at immediate post-, and 8-week post-training using Google Forms to measure [v] skill confidence in basic cardiac ultrasound, [vi] knowledge confidence in basic cardiac ultrasound, and [vii] overall curriculum satisfaction.

### Curriculum feasibility criteria

We semi-quantitatively evaluated the feasibility of the curriculum in terms of recruitment rate, follow-up rate 8 weeks after training, instructional design of the hands-on training session, the effect size (ES) of the test score improvements, and participant satisfaction. Feasibility criteria for recruitment rate and follow-up rate 8 weeks after training were 80% or more. We subjectively assessed the feasibility of the instructional design as not feasible, feasible with modifications, or feasible as is [18]. Identified instructional design flaws were modified where possible. We utilized student curriculum feedback to guide modifications. With respect to the ES of the test score improvements, the criteria were that the ES of the 4 skill and knowledge test score improvements [i–iv] were moderate or large (ES greater than 0.5). With regard to participant satisfaction, the criteria were that the students report satisfaction with the overall curriculum [vii] at post- and 8-week post-training, with mean 5-point Likert scale ratings greater than 3. Based on these feasibility outcomes, we evaluated the overall curriculum as not feasible, feasible with modifications, or feasible as is to implement for a definitive study.

### Sample size

The main focus of this study was to develop the cardiac POCUS curriculum with test scoring systems, and then pilot test it on a small sample size of 6 participants to confirm the curriculum feasibility and gain feedback before progressing to a larger scale study. Numerical data of this study could be utilized for sample size estimation for a future definitive study, but the accuracy of the estimation is limited because of the small samples [29].

### Statistical analysis

All statistical analyses were performed using BellCurve for Excel (Social Survey Research Information Co., Ltd.). All numeric variables are presented as mean ± SD. Mean difference between pre- and immediate post- or 8-week post-training data were calculated with paired *t*-test and presented as mean and SD with 95% confidence interval (CI) and the ES. We interpreted the clinical significance of ES according to Cohen’s effect size guidelines (0.2 to 0.5 = small ES, 0.5 to 0.8 = moderate ES, and more than 0.8 = large ES) [30, 31]. ICC estimates and their 95% confidence intervals were calculated based on a mean rating (*k* = 3), absolute-agreement, 2-way random-effects models for interrater reliability, and a mean rating (*k* = 2), absolute-agreement, 2-way mixed-effects models for test-retest reliability. We interpreted the ICC estimates according to ICC reporting guidelines (ICC of less than 0.5 = poor, 0.5 to 0.75 = moderate, 0.75 to 0.9 = good, and greater than 0.9 = excellent reliability) [32]. Due to the small sample size of 6 participants, all numerical data should be treated with caution and treated as preliminary.

## Results

### Characteristics of participants

The first 6 second-year medical students we approached agreed to participate in this study. Student demographic data and previous ultrasound training experience are shown in Table 2. Three students attended an unstructured 1-h introductory focused assessment with sonography in trauma (FAST) hands-on training using student volunteers during a first-year medical school elective before participation in this study. No students had ultrasound experience on patients or simulators. Between the immediate post-training test and the 8-week post-training test, 3 students reviewed relevant textbooks or websites and 1 student participated in a FAST examination hands-on training on a student volunteer with the medical school ultrasound interest group.

**Table 2:** Characteristics of participants

	Student A	Student B	Student C	Student D	Student E	Student F
School year	2nd	2nd	2nd	2nd	2nd	2nd
Sex	Male	Male	Male	Female	Male	Female
Dominant hand	Right	Right	Right	Right	Right	Right
Group size in hand-on training session	3-student group	3-student group	3-student group	1-student group	2-student group	2-student group
Pre-training skill test score (10-point maximum)	2.67	2	3.67	4.33	2.33	0.33
Pre-training knowledge test score (40-point maximum)	n/a^a^	14	29	18	17	20
Previous ultrasound training experience						
Ultrasound hands-on training or lecture	No	No	Yes^b^	Yes^b^	Yes^b^	No
Cardiac ultrasound on patients	No	No	No	No	No	No
Observation of a cardiac ultrasound on patients	Yes (1–4 times)	No	No	No	Yes (1–4 times)	Yes (1–4 times)
Cardiac ultrasound on healthy volunteers	Yes	No	Yes (1 time)^b^	Yes (1 time)^b^	Yes (1 time)^b^	No
Cardiac ultrasound on simulators	No	No	No	No	No	No
Completion of pre-training ASE online module self-study	Yes	Yes	Yes	Yes	Yes	Yes
ASE online module review between immediate post-training tests and 8-week post-training tests	No	No	No	No	No	No
Review of textbooks or websites other than ASE online module between immediate post-training tests and 8-week post-training tests	No	No	No	Yes	Yes	Yes
Additional hands-on training between immediate post-training tests and 8-week post-training tests	No	No	No	Yes^c^	No	No

*n/a*, not available; *ASE*, American Society of Echocardiography; *FAST*, Focused Assessment with Sonography in Trauma

^a^Student A did not complete the pre-training knowledge test

^b^Students attended an unstructured 1-h introductory FAST hands-on training with student volunteers in a medical school elective before participation in this study

^c^Student D attended a FAST hands-on training with student volunteers in a medical school ultrasound interest group between the immediate post-training test and the 8-week post-training test

### Curriculum learning effect outcomes

**Fig. 3:**
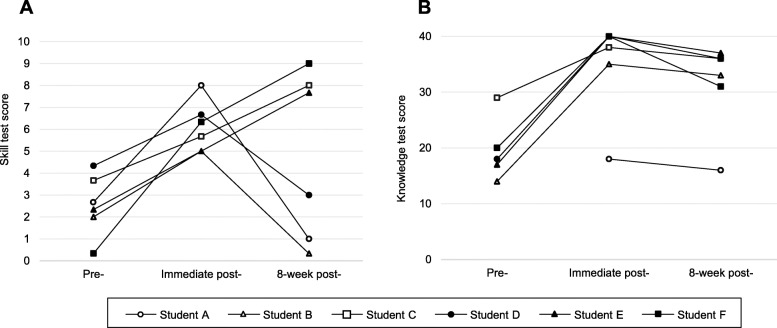
Individual skill (**A**) and knowledge (**B**) test scores

All 6 students completed the pre-training ASE online module self-study, the hands-on training session, all the skill tests, and the post-training 5-point Likert scale questionnaires. Five students completed all the knowledge tests; one student did not complete the pre-training knowledge test. Individual skill and knowledge test scores are illustrated in Fig. 3, and mean skill test scores and the scores for each of the 5 views are summarized in Table 3.

**Table 3:** Mean skill test scores and the scores for each of 5 cardiac POCUS views

	Pre-training	Immediate post-training	8-week post-training
Skill test score^a^	2.56 ± 1.40	6.11 ± 1.15	4.83 ± 3.84
PLAX score^b^	0.89 ± 1.00	1.06 ± 0.71	1.22 ± 0.96
PSAX score^b^	0.44 ± 0.40	1.06 ± 0.14	0.50 ± 0.55
A4C score^b^	0.67 ± 0.70	0.89 ± 0.86	1.00 ± 0.87
S4C score^b^	0.56 ± 0.58	1.11 ± 0.50	1.11 ± 0.94
SIVC score^b^	0.00 ± 0.00	2.00 ± 0.00	1.00 ± 1.10

*PLAX*, parasternal long-axis view; *PSAX*, papillary muscle level of parasternal short-axis view; *A4C*, apical 4-chamber view; *S4C*, subcostal 4-chamber view; *SIVC*, subcostal inferior vena cava view

^a^10-point maximum score, ^b^2-point maximum score

Data are presented as mean ± SD

Note: The 2-point maximum scores for each of 5 cardiac POCUS views are added for the 10-point maximum skill test score

#### Skill test score

[i] Mean skill test score difference between pre-training and immediate post-training was 3.56 points (SD, 1.68; 95% CI, 1.79 to 5.32; large ES of 3.05) and [ii] the difference between pre-training and 8-week post-training was 2.28 points (SD, 4.44; 95% CI, − 2.38 to 6.94; large ES of 0.86) (Fig. 3A). Interrater reliability of the skill test scoring system assessed using all 18 score results of 10-point maximum skill tests from the 6 students was excellent (ICC, 0.96; 95% CI, 0.88 to 0.98). The interrater reliability assessed using all 90 score results of 2-point maximum scores from each of 5 views was excellent (ICC, 0.91; 95% CI, 0.87 to 0.94). Test-retest reliability of the 3 raters using re-assessed 18 score results of 10-point maximum skill tests was excellent (ICC, 0.97; 95% CI, 0.63 to 0.99). The test-retest reliability using re-assessed 90 score results of 2-point maximum scores from each of 5 views was excellent (ICC, 0.94; 95% CI, 0.90 to 0.97).

#### Knowledge test score

[iii] Mean knowledge test score difference between pre-training and immediate post-training was 19.0 points (SD, 5.7; 95% CI, 11.9 to 26.1; large ES of 4.93) and [iv] the difference between pre-training and 8-week post-training was 15.0 points (SD, 5.7; 95% CI, 7.9 to 22.0; large ES of 3.82) (Fig. 3B).

#### Post-training questionnaire

Mean 5-point Likert rating scale results at immediate post- and 8-week post-training were that [v] skill confidence in basic cardiac ultrasound were 4.0 ± 0.6 and 3.8 ± 1.0, [vi] knowledge confidence in basic cardiac ultrasound were 4.0 ± 0.6 and 3.5 ± 1.2, and [vii] overall curriculum satisfaction were 4.5 ± 0.5 and 4.5 ± 0.5 (mean ± SD).

### Curriculum feasibility

The recruitment rate was 100% (6/6 students) and follow-up rate 8 weeks after training was 100% (6/6 students). We identified several instructional design flaws requiring modification of the hands-on training group size, content of the cardiac POCUS lecture, hands-on teaching instructions, and HHU usage. The instructional design modifications are detailed in the discussion section.

The 4 skill and knowledge pre-post learning effects ([i] skill test score difference between pre-training and immediate post-training, [ii] the difference between pre-training and 8-week post-training, [iii] knowledge test score difference between pre-training and immediate post-training and [iv] the difference between pre-training and 8-week post-training) each demonstrated a large ES of 3.05, 0.86, 4.93, and 3.82, respectively. The students reported [vii] overall curriculum satisfaction with mean 5-point Likert scale ratings were greater than 3 points; 4.5 ± 0.5 at post-training and 4.5 ± 0.5 at 8-week post-training (mean ± SD). Based on these feasibility outcomes, we evaluated the overall curriculum’s feasibility as feasible with modifications to implement the future definitive study.

## Discussion

This pilot study confirmed the feasibility of the pre-clinical medical student cardiac POCUS curriculum, without recruitment and attrition concerns, with large ES of learning effect outcomes, and favorable student satisfaction with the curriculum. We identified several curriculum design flaws that required modification including the hands-on training group size, content of the cardiac POCUS lecture, the hands-on teaching instructions, and HHU usage.

### Curriculum design modifications

#### Hands-on training group size

This pilot study trialed hands-on training group sizes of 3 students, 2 students, and 1 student to determine a feasible group size for a definitive study. In the 3-student group size, hands-on training required an excess of 20 min beyond the programmed training time to provide adequate hands-on practice for each student. Moreover, each of the students had 40 min additional waiting time while other students took the pre- and immediate post-training skill tests. We deemed this long waiting time was inappropriate and impractical for volunteer participants. Both the 2-student group and the 1-student group did not raise concerns regarding time management and efficiency. However, during the 2-student group session, we observed the potential for positive or negative peer interactions affecting learning experiences, which could lead to intervention heterogeneity within or between groups, and interfere with consistency and continuity of individualized instructor feedback [33]. A study that compared constant instructor feedback to intermittent feedback with peer feedback in chiropractic skill training demonstrated that constant feedback was advantageous for accurate skill acquisition and initial hands-on practice, while the intermittent feedback was advantageous for long-term skill retention [34]. A study of cultural differences in simulation-based learning showed an individual learner’s interaction pattern with instructors and colleagues during debriefing could affect learning effectiveness [35]. These studies suggest a multiple student group size may affect student learning and lead to intervention heterogeneity within a group or between groups. In addition, we speculated that recruitment of more than 1 volunteer for a single session could be difficult considering medical student schedules. Based on the pilot study observations, we selected the 1-student group size with no waiting time, less intervention heterogeneity, and ease of scheduling with student volunteers for the definitive study.

This study did not intend to determine an ideal or practical group size. The choice of 1-student group size was for feasibility to conduct a future study investigating accurate and unbiased learning effects, not to reveal the optimal group size for learning effectiveness or efficiency. This study also did not seek to identify a realistic or practical group size for curriculum integration into the class size of about 70 students per year in our medical school. We assume it is impractical to implement 1-on-1 sessions for all. At present, there is no evidence regarding an ideal or practical group size for cardiac POCUS training [15]. The *Teaching the Teacher (Module E)* of the ASE online module recommends a ratio of 4 students to 1 healthy volunteer to 1 instructor as the ideal learning environment (https://aselearninghub.org/). The American Heart Association (AHA) basic life support (BLS) course may provide guidance regarding a practical group size. The BLS course recommends a ratio of 6 students to 2 manikins to 1 instructor which allows instructors to observe the students, provide feedback and guide the students’ acquisition of skills [36]. These recommendations may serve as important references for medical school ultrasound training curriculum integration.

#### Content of cardiac POCUS lecture

Based on student curriculum feedback, we modified the content of the cardiac POCUS lecture for the definitive study. In this pilot study, we included didactic teaching on the cardiac ultrasound beam shape, probe orientation marker, and probe manipulation during the hands-on training, since the pre-training ASE online modules (A, B, and C) did not provide content on these topics. We also included a question-and-answer review of anatomical structures seen in the 5 views during the hands-on training to confirm and reinforce student knowledge. Two students gave feedback that these didactic contents would be better provided during the lecture rather than during the hands-on training in order to confirm prerequisite knowledge before hands-on practice and focus on manual skill acquisition during the hands-on training time. In the definitive study, we plan to incorporate the items not involving direct hands-on practice into the cardiac POCUS lecture for effective use of hands-on training time. The modified content of the cardiac POCUS lecture is in Additional File 6. Although mentioned in the ASE online module, all 6 students did not understand that the papillary muscle level of parasternal short-axis view was helpful for left ventricle (LV) wall motion analysis. We plan to reinforce this clinically important concept in the definitive study.

#### 5-view hands-on teaching instructions

Mean skill test scores for PSAX at 8-week post-training and A4C score at immediate post-training were less than 1.0 point, which means poor or unacceptable for clinical use (Table 3). We plan to modify the PSAX teaching instruction with reference to a POCUS textbook [27] in a more understandable way with step-by-step instruction of probe manipulation using the script:

First, you start from a clear image of PLAX. Second, you rotate the probe 90 degrees clockwise, and then you usually find the fish mouth appearance of the mitral valve level in the short-axis view. Third, you tilt the probe downward carefully until you see 2 papillary muscles and you should get a clear image of the papillary muscle level of the parasternal short-axis view.

During A4C teaching in the study, in order to demonstrate a true longitudinal LV cavity, we instructed students to tilt up a probe adequately after placing the probe on an intercostal space without distorting the image. However, we observed that distorted images after adequate probe tilting was unavoidable for the healthy volunteer A4C image acquisition with the HHU. Therefore, we accepted a mild LV foreshortening and plan to prioritize clear visualization of all 4 chambers in order to provide straightforward instructions to novice students for the definitive study. Our modification of the A4C teaching instruction was supported by ASE POCUS and comprehensive TTE guidelines [3, 26].

The SIVC teaching instruction resulted in excellent quality image acquisition by all students immediately after training (Table 3). However, all students queried the significance of SIVC findings in clinical practice. In our curriculum, we did not focus on image interpretation but rather on proper image acquisition for initial training in cardiac POCUS skills for novice students at an appropriate level for learning effectiveness [15, 20]. In order to balance student curiosity with maintaining an appropriate difficulty level for the curriculum, we plan to provide a brief commentary about volume responsiveness assessment and central venous pressure estimation using inferior vena cava (IVC) diameter based on our clinical experience, ASE TTE guidelines [26, 37], and a POCUS textbook [27].

#### HHU (Butterfly iQ) limitations

We experienced automatic ultrasound scan shut-off due to probe temperature increase after continuous scanning during the hands-on training. Once the probe heated up and shut down, we had to suspend scanning for several minutes until the probe cooled down [28]. To manage this technical issue, we plan to utilize 2 Butterfly iQ probes in the hands-on training session for the definitive study.

### Test scoring system validity evidence

We gathered validity evidence based on Messick’s framework recommended by the American Educational Research Association (AERA) [38]. AHA also referenced Messick’s framework to evaluate assessment tools for resuscitation in their scientific statement of resuscitation education science [39]. Messick’s framework classifies sources of validity evidence into 5 categories: *content*, *response process*, *internal structure*, *relations to other variables*, *and consequences*. In our study, *content evidence* refers to the relationship between the test content and what is intended to measure; *response process evidence* assesses test quality control or the extent to which the test administration and scoring are controlled or standardized to reflects the observed performance; *internal structure evidence* evaluates the reliability and reproducibility of the test results; *relations to other variables evidence* evaluates discriminatory ability of the test between experts and novices or the correlation between the test results and known measures of competence; and *consequences evidence* refers to downstream effects of test results and the decisions that are made [40–42]. Our skill and knowledge test scoring systems were developed by echocardiography experts, through extensive review and evaluation of previously published studies, expert discussions, and an iterative design process, thus contributing to the *content evidence*. Our control measures for obtaining the ultrasound video clips involved asking the students to record when they perceived they had obtained their “best” view, allowed for a second chance to record another view, and had the student choose which of the 2 recordings to submit for assessment. The students controlled the recording, without instructor intervention, therefore adding to the *response process evidence* of the skill scoring system. Our skill test scoring system demonstrated excellent interrater reliability and test-retest reliability which contributes to the *internal structure evidence* of this tool. With regard to *relations to other variables evidence*, we did not assess the test scoring system discriminatory ability between experts and novices for echocardiography because of small novice samples. We planned to gather the evidence in the definitive study with sufficient novice samples. With respect to *consequences evidence*, we did not gather the evidence because our pilot study did not intend to establish a pass/fail cut-off score or identify the clinical impact of the curriculum. Given the evidence for validity demonstrated by our tools, we determine that these scoring systems can be utilized in the definitive study.

### Post-8-week skill retention

Skill test score results demonstrated that 3 students (student C, E, and F) retained skill 8 weeks after training and that 3 (student A, B, and D) did not (Fig. 3). From the post-training questionnaires, we determined 2 possible reasons for the inconsistent skill retention finding. Of the 3 students who retained skill, 2 (student C and E) attended an unstructured 1-h introductory FAST hands-on training elective using student volunteers before the participation in this study and 2 (student E and F) reviewed relevant websites or textbooks after hands-on training (Table 2). We cannot reach decisive conclusions about the divergent skill retention finding due to the small pilot study sample size. If the definitive study demonstrates similar findings, we plan to formally examine demographic factors that could explain individual skill retention variation. We also found that 8-week post-training skill test scores in the 3 (student A, B, and D) who did not retain their skill were lower than their pre-training scores. One possible reason for the lower scores is that the pre-training skill test was performed after pre-training self-study of the ASE online module, so the test scores did not necessarily represent a true baseline but rather skill after a recent review of the online module content. The 3 students may not have retained the online module content in addition to the skills acquired during hands-on training.

### Single primary outcome selection for a definitive study

In this pilot study, we measured 7 learning effect outcomes. We needed to select the most important single primary outcome measure or question in order to establish a primary hypothesis for the definitive study. Our pilot study demonstrated short-term knowledge and skill retention and long-term knowledge retention with a large ES and the 95% CI not including zero, with consistently favorable student confidence and the curriculum satisfaction ratings. However, the skill retention remains unknown due to the small sample size, even though the ES of the skill retention was large. Most previous studies investigating the learning effect of cardiac ultrasound curriculum showed psychomotor or cognitive skill outcomes were improved immediately after an initial training [10, 13, 14, 43–50]. Assessments in these studies focused on very short-term knowledge and/or skill retention. Several studies have demonstrated long-term knowledge or confidence retention after several weeks or months [22, 25, 51, 52], however, few studies have investigated long-term skill retention [22]. The importance of investigating long-term skill retention is evident both from the results of our pilot and our review of the literature. Therefore, we selected the outcome of the skill test score difference between pre-training and 8-week post-training as a single primary outcome for the definitive study that is registered in ClinicalTrials.gov (Identifier: NCT04083924).

## Conclusion

This pilot study confirmed the feasibility of the ASE-recommended medical student cardiac POCUS curriculum, without recruitment and attrition concerns, with large ESs of skill and knowledge learning effect outcomes, and favorable student curriculum satisfaction. The skill and knowledge test scoring systems gathered validity evidence. We modified several curriculum design areas including the hands-on training group size, cardiac POCUS lecture content, the hands-on teaching instructions, and HHU usage. Based on the pilot study findings, we plan to conduct the definitive study with the primary outcome of the long-term skill retention 8 weeks after initial training.

## Supplementary Information

**Additional file 1.** CONSORT 2010 checklist of information to include when reporting a pilot or feasibility trial.

**Additional file 2.** Original content of cardiac POCUS lecture.

**Additional file 3.** Main instruction points for 5 cardiac POCUS views image acquisition.

**Additional file 4.** Skill test scoring system development in detail.

**Additional file 5.** 40-point maximum knowledge test scoring system.

**Additional file 6.** Modified content of cardiac POCUS lecture.

## Data Availability

The datasets used and/or analyzed during the current study are available from the corresponding author on reasonable request.

## Abbreviations

AERA: American Educational Research Association
AHA: American Heart Association
ASE: American Society of Echocardiography
A4C: Apical 4-chamber view
BLS: Basic life support
CCE: Critical care echocardiography
CI: Confidence interval
CONSORT: Consolidated Standards of Reporting Trials
EACVI: European Association of Cardiovascular Imaging
ES: Effect size
FAST: Focused assessment with sonography in trauma
FEEL: Focused Echocardiography Entry Level
FoCUS: Focused cardiac ultrasound
HHU: Hand-held ultrasound
ICC: Intraclass correlation coefficient
IVC: Inferior vena cava
JABSOM: John A. Burns School of Medicine
LV: Left ventricle
PLAX: Parasternal long-axis view
POCUS: Point-of-care ultrasound
PSAX: Papillary muscle level of parasternal short-axis view
SD: Standard deviation
SIVC: Subcostal inferior vena cava view
S4C: Subcostal 4-chamber view
TTE: Transthoracic echocardiography
UME: Undergraduate medical education
WINFOCUS: World Interactive Network Focused on Critical UltraSound
