# Supplementary material for: Pre-clinical medical student cardiac point-of-care ultrasound curriculum based on the American Society of Echocardiography recommendations: a pilot and feasibility study
Source: Pilot Feasibility Stud. 2021 Sep 14;7:175. doi: 10.1186/s40814-021-00910-3 (PMC8438804; doi:10.1186/s40814-021-00910-3)
Supplement: Supplementary file 4 — Additional file 4. Skill test scoring system development in detail. [file 40814_2021_910_MOESM4_ESM.doc]

**Additional File 4: Skill test scoring system development in detail**

We developed a 10-point maximum skill test scoring system by modifying an existing assessment tool for transthoracic echocardiography (TTE) views [1]. We also referenced the American Society of Echocardiography (ASE) comprehensive TTE guidelines [2] to elect the anatomical structures to demonstrate in the 5 cardiac point-of-care ultrasound (POCUS) views. The scoring system was designed to assess 5-view image quality for the purpose of rapid bedside cardiac assessment, not for a formal diagnostic comprehensive echocardiography examination. While TTE image quality scoring systems exist, to our knowledge, there is no standardized or validated scoring system to assess image quality of cardiac POCUS or TTE views. There is one validated scoring system to evaluate image quality of transesophageal echocardiography (TEE) views [3,4]. Initially, the first author (SJ) modified the validated TEE scoring system to devise an image quality scoring system for the 5 cardiac POCUS views. He further discussed the modifications from a TEE to a TTE image quality assessment tool with the 3 cardiologists (AK, MI, and KK) specializing in echocardiography and echocardiography education in their university hospitals. However, the 3 cardiologists judged the substitution of TEE scoring system for TTE’s to be unsuitable due to the inherent image clarity difference between TEE and TTE [2,5]. In general, TEE image clarity is better than TTE because TEE images can be obtained through the esophagus without obstacles like ribs or lungs that TTE has. Therefore, we decided to utilize existing TTE assessment tools for the 5-view image quality scoring system in our study.

Most existing TTE view assessment tools are subjective or qualitative scoring systems and the selected views for the assessment varied in each of the studies where they were used [6,7,16,8–15]. However, one previous study [1] utilized an objective, quantitative, and reliable assessment tool for the same 5 views as in our study. The assessment tool was a 15-point maximum test scoring system that was designed to test the ability to acquire clips with sufficient quality to be useful for assessment of hemodynamic status. The 15-point maximum test scoring system rated the 5 views; each received a score ranging from 0-3 points. Each view was scored 3 points (similar to “gold standard” quality), 2 points, 1 point (poor quality), or 0 point (structures not recognizable) for POCUS use. The scoring system demonstrated “almost-perfect agreement” between 2 raters using the Krippendorff test for inter-rater reliability; the alpha coefficient was 0.85 (95% CI 0.81 to 0.88). This scoring system had almost the same content as we intend to measure, which was image acquisition skill for a cardiac POCUS rather than for a formal diagnostic comprehensive echocardiography examination. Thus, the cardiologists agreed that this tool would be the basis for designing our own assessment tool after several modifications. The most significant modification was the maximum score for each of the 5 views. In the original scoring system, each view received a score ranging from 0-3 points. The criteria of 0 point was “structures not recognizable”, while the 1 point “poor quality”. For example, the “poor quality” for parasternal short-axis view was defined that “both left ventricle and right ventricle not visualized properly”. We considered that the criteria differences between 0 and 1 point did not translate into clinically meaningful differences and that it could lead to an overestimation of image quality improvement after training. Therefore, we modified the scoring system so that each view received a score ranging from 0-2 points and the maximum score range for all 5 views would be 0-10 points. In the 10-point maximum skill test scoring system, each view was assessed as excellent (2 points), acceptable (1 point), or poor (0 point) for cardiac POCUS use (See Table 1 for detailed image quality criteria).

**References**

1. Edrich T, Seethala RR, Olenchock BA, Mizuguchi AK, Rivero JM, Beutler SS, et al. Providing initial transthoracic echocardiography training for anesthesiologists: simulator training is not inferior to live training. J Cardiothorac Vasc Anesth. 2014;28:49–53.

2. Mitchell C, Rahko PS, Blauwet LA, Canaday B, Finstuen JA, Foster MC, et al. Guidelines for performing a comprehensive transthoracic echocardiographic examination in adults: recommendations from the American Society of Echocardiography. J Am Soc Echocardiogr. 2019;32:1–64.

3. Ferrero NA, Bortsov A V., Arora H, Martinelli SM, Kolarczyk LM, Teeter EC, et al. Simulator training enhances resident performance in transesophageal echocardiography. Anesthesiology. 2014;120:149–59.

4. Bloch A, von Arx R, Etter R, Berger D, Kaiser H, Lenz A, et al. Impact of Simulator-Based Training in Focused Transesophageal Echocardiography: A Randomized Controlled Trial. Anesth Analg. 2017;125:1140–8.

5. Hahn RT, Abraham T, Adams MS, Bruce CJ, Glas KE, Lang RM, et al. Guidelines for performing a comprehensive transesophageal echocardiographic examination: recommendations from the American Society of Echocardiography and the Society of Cardiovascular Anesthesiologists. Anesth Analg. 2014;118:21–68.

6. Neelankavil J, Howard-Quijano K, Hsieh TC, Ramsingh D, Scovotti JC, Chua JH, et al. Transthoracic echocardiography simulation is an efficient method to train anesthesiologists in basic transthoracic echocardiography skills. Anesth Analg. 2012;115:1042–51.

7. Cawthorn TR, Nickel C, O’Reilly M, Kafka H, Tam JW, Jackson LC, et al. Development and evaluation of methodologies for teaching focused cardiac ultrasound skills to medical students. J Am Soc Echocardiogr. 2014;27:302–9.

8. Ho AMH, Critchley LAH, Leung JYC, Kan PKY, Au SS, Ng SK, et al. Introducing final-year medical students to pocket-sized ultrasound imaging: teaching transthoracic echocardiography on a 2-week anesthesia rotation. Teach Learn Med. 2015;27:307–13.

9. Wilkinson JS, Barake W, Smith C, Thakrar A, Johri AM. Limitations of condensed teaching strategies to develop hand-held cardiac ultrasonography skills in internal medicine residents. Can J Cardiol. 2016;32:1034–7.

10. Kusunose K, Yamada H, Suzukawa R, Hirata Y, Yamao M, Ise T, et al. Effects of transthoracic echocardiographic simulator training on performance and satisfaction in medical students. J Am Soc Echocardiogr. 2016;29:375–7.

11. Nelson BP, Hojsak J, Dei Rossi E, Karani R, Narula J. Seeing is believing: evaluating a point-of-care ultrasound curriculum for 1st-year medical students. Teach Learn Med. 2017;29:85–92.

12. Kobal SL, Lior Y, Ben-Sasson A, Liel-Cohen N, Galante O, Fuchs L. The feasibility and efficacy of implementing a focused cardiac ultrasound course into a medical school curriculum. BMC Med Educ. 2017;17:94.

13. Fuchs L, Gilad D, Mizrakli Y, Sadeh R, Galante O, Kobal S. Self-learning of point-of-care cardiac ultrasound - Can medical students teach themselves? PLoS One. 2018;13:e0204087.

14. Kumar A, Barman N, Lurie J, He H, Goldman M, McCullough SA. Development of a point-of-care cardiovascular ultrasound program for preclinical medical students. J Am Soc Echocardiogr. 2018;31:1064-1066.e2.

15. Chaptal M, Tendron L, Claret P-G, Muller L, Markarian T, Mattatia L, et al. Focused cardiac ultrasound: a prospective randomized study of simulator-based training. J Am Soc Echocardiogr. 2020;33:404–6.

16. Andersen GN, Viset A, Mjølstad OC, Salvesen O, Dalen H, Haugen BO. Feasibility and accuracy of point-of-care pocket-size ultrasonography performed by medical students. BMC Med Educ. 2014;14:156.
