# Supplementary material for: Pre-clinical medical student cardiac point-of-care ultrasound curriculum based on the American Society of Echocardiography recommendations: a pilot and feasibility study
Source: Pilot Feasibility Stud. 2021 Sep 14;7:175. doi: 10.1186/s40814-021-00910-3 (PMC8438804; doi:10.1186/s40814-021-00910-3)
Supplement: Supplementary file 5 — Additional file 5. 40-point maximum knowledge test scoring system. [file 40814_2021_910_MOESM5_ESM.pdf]

Additional File 5

5 cardiac POCUS views knowledge test (40-point maximum)

This knowledge test includes 40 multiple-choice questions; (Part 1: 9 questions, Part 2: 10 questions, Part 3: 9 questions, Part 4: 7 questions, Part 5: 5 questions).
Please choose correct answer from the answer choices below.

Please complete the test within 30 minutes.

Please do not review any resources such as textbooks or websites prior to or during your knowledge assessment.

Answer choices

1. Right Atrium
2. Right Atrial Appendage
3. Right Ventricle
4. Left Atrium
5. Left Atrial Appendage
6. Left Ventricle
7. Aortic Valve
8. Mitral Valve
9. Tricuspid Valve
10. Pulmonary Valve
11. Ascending Aorta
12. Descending Aorta
13. Aortic Arch
14. Pulmonary Artery
15. Pulmonary Vein
16. Superior Vena Cava
17. IVC
18. Interatrial Septum
19. Interventricular Septum
20. Anterior wall of LV
21. Inferior wall of LV
22. Lateral wall of LV (Antero-lateral)
23. Antero-lateral Papillary Muscle
24. Postero-medial Papillary Muscle
25. Diaphragm
26. Pericardium
27. Liver
28. Lung
29. Kidney
30. Spleen
31. Parasternal long-axis view
32. Parasternal short-axis view
33. Apical 4-chamber view
34. Apical 2-chamber view
35. Apical 3-chamber view
36. Apical 5-chamber view
37. Suprasternal view
38. Subcostal 4-chamber view
39. Subcostal IVC view
40. I have no idea.

IVC = Inferior Vena Cava, LV = Left Ventricle

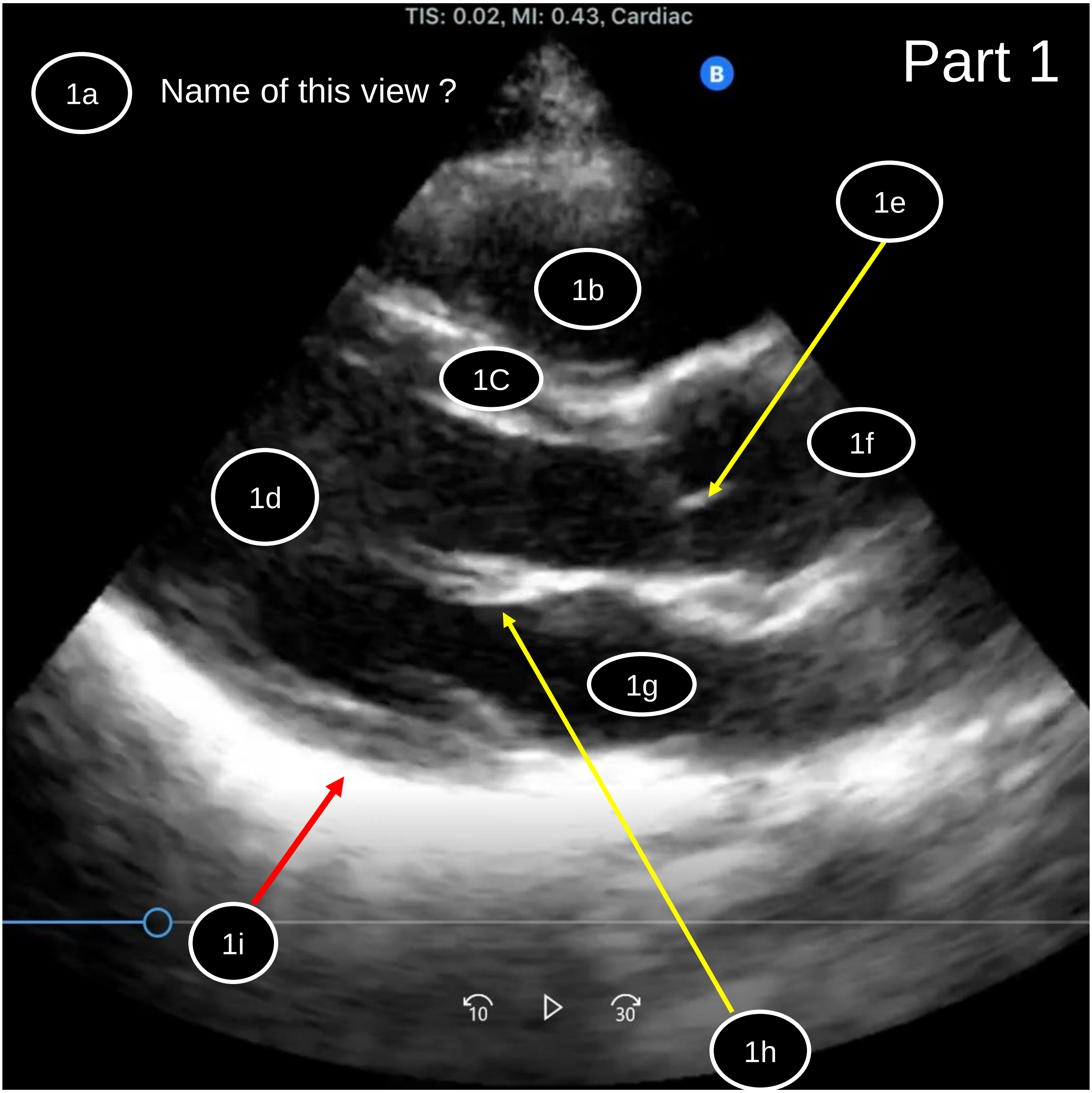

Question 1-1. What is the name of view **1a** ?
Question 1-2. What is **1b** ?
Question 1-3. What is **1c** ?
Question 1-4. What is **1d** ?
Question 1-5. What is **1e** ?
Question 1-6. What is **1f** ?
Question 1-7. What is **1g** ?
Question 1-8. What is **1h** ?
Question 1-9. What is the brighter region **1i** ?

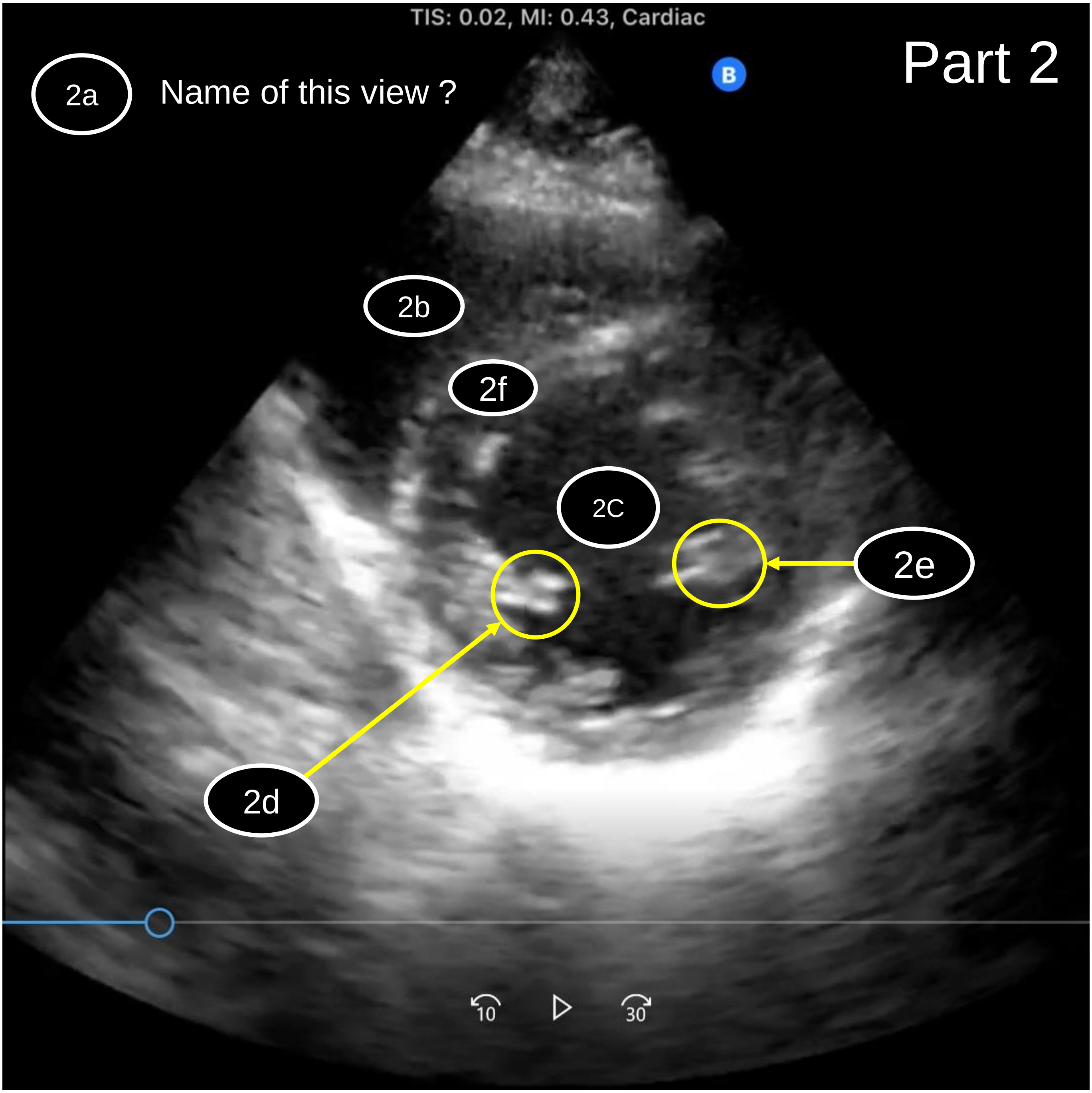

Question 2-1. What is the name of view **2a** ?
Question 2-2. What is **2b** ?
Question 2-3. What is **2c** ?
Question 2-4. What is **2d** ?
Question 2-5. What is **2e** ?
Question 2-6. What is **2f** ?

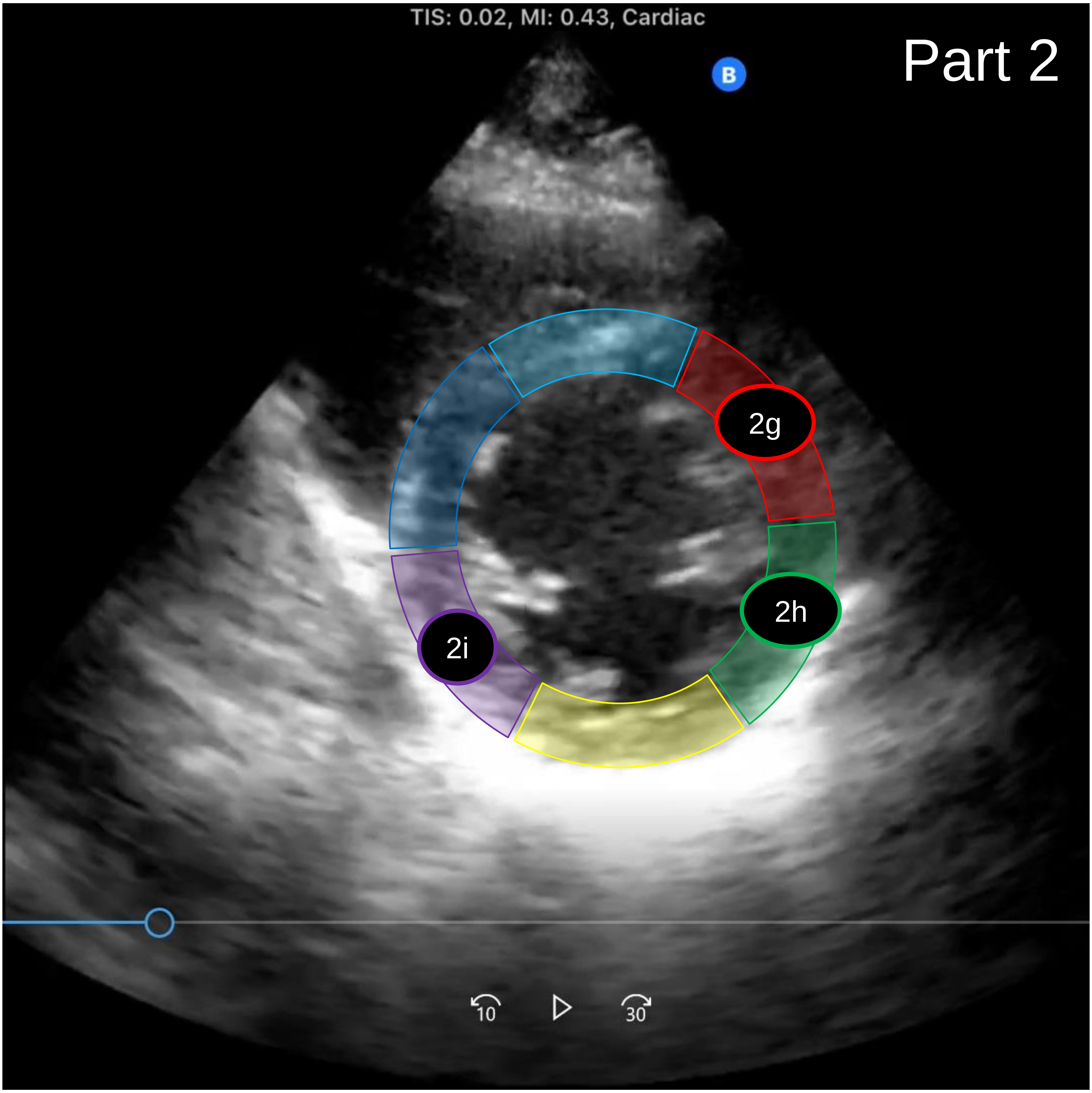

Question 2-7. What segment of left ventricle wall is **2g** ?
Question 2-8. What segment of left ventricle wall is **2h** ?
Question 2-9. What segment of left ventricle wall is **2i** ?

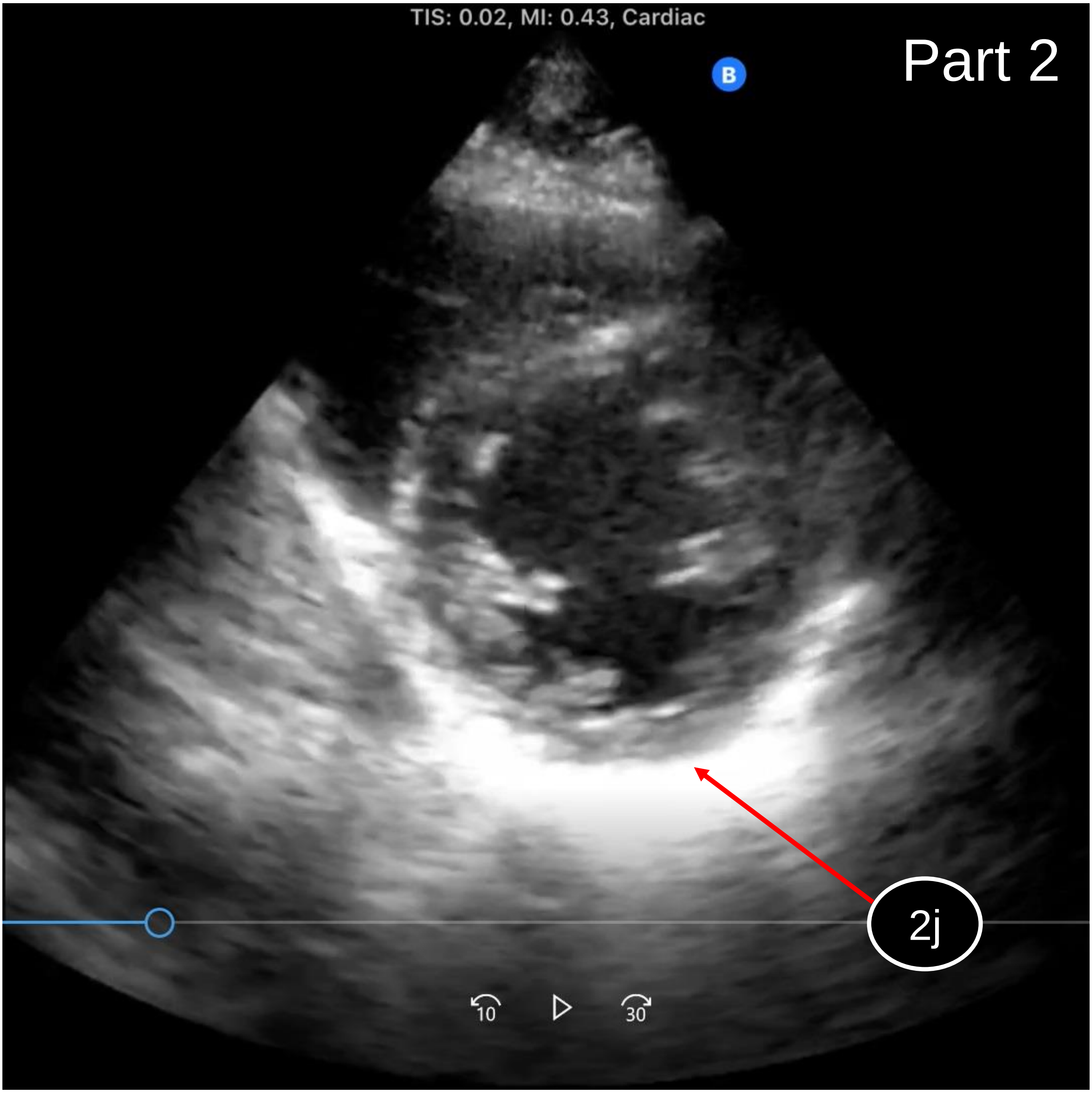

Question 2-10. What is the brighter region **2j** ?

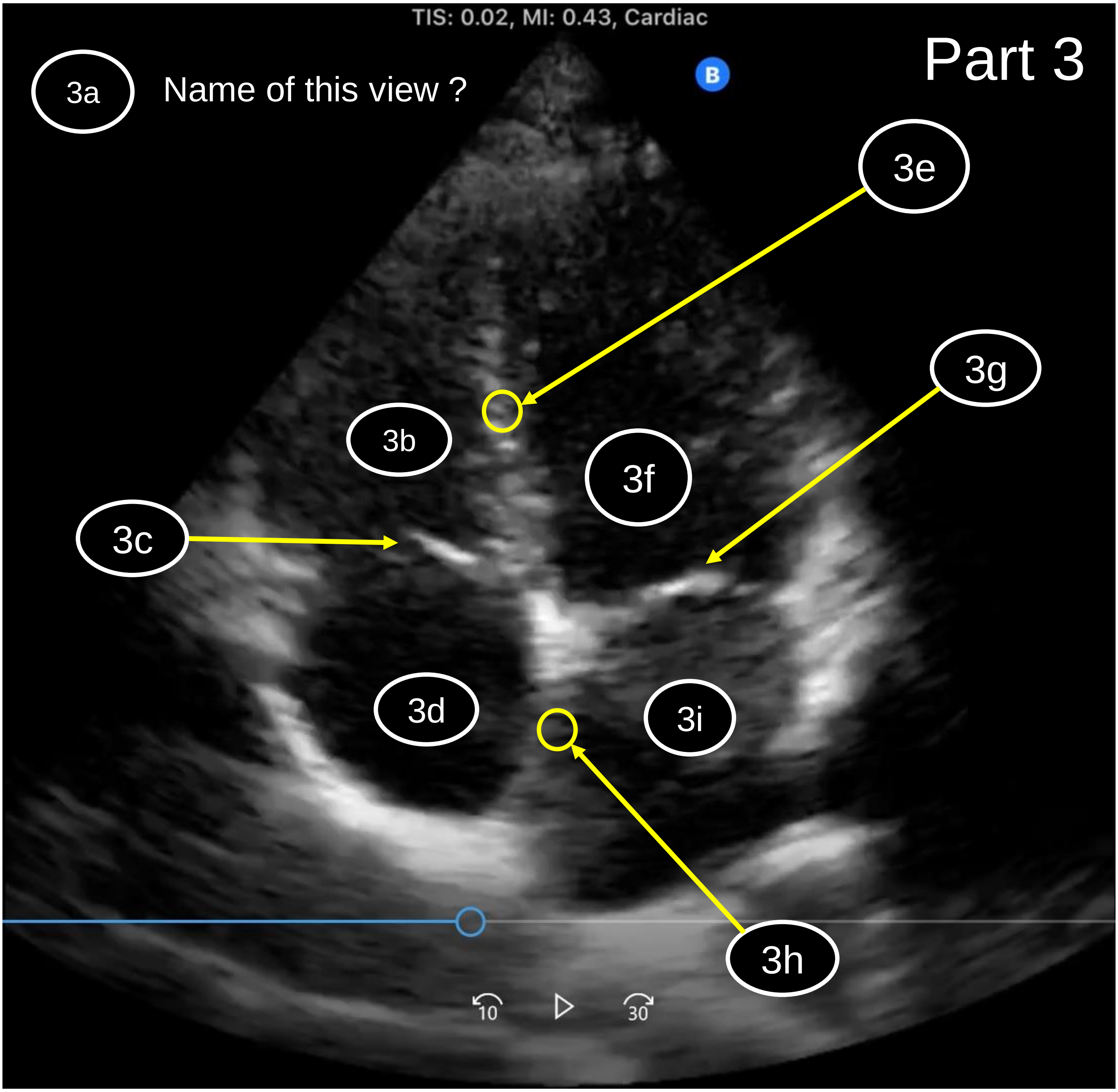

Question 3-1. What is the name of view **3a** ?
Question 3-2. What is **3b** ?
Question 3-3. What is **3c** ?
Question 3-4. What is **3d** ?
Question 3-5. What is **3e** ?
Question 3-6. What is **3f** ?
Question 3-7. What is **3g** ?
Question 3-8. What is **3h** ?
Question 3-9. What is **3i** ?

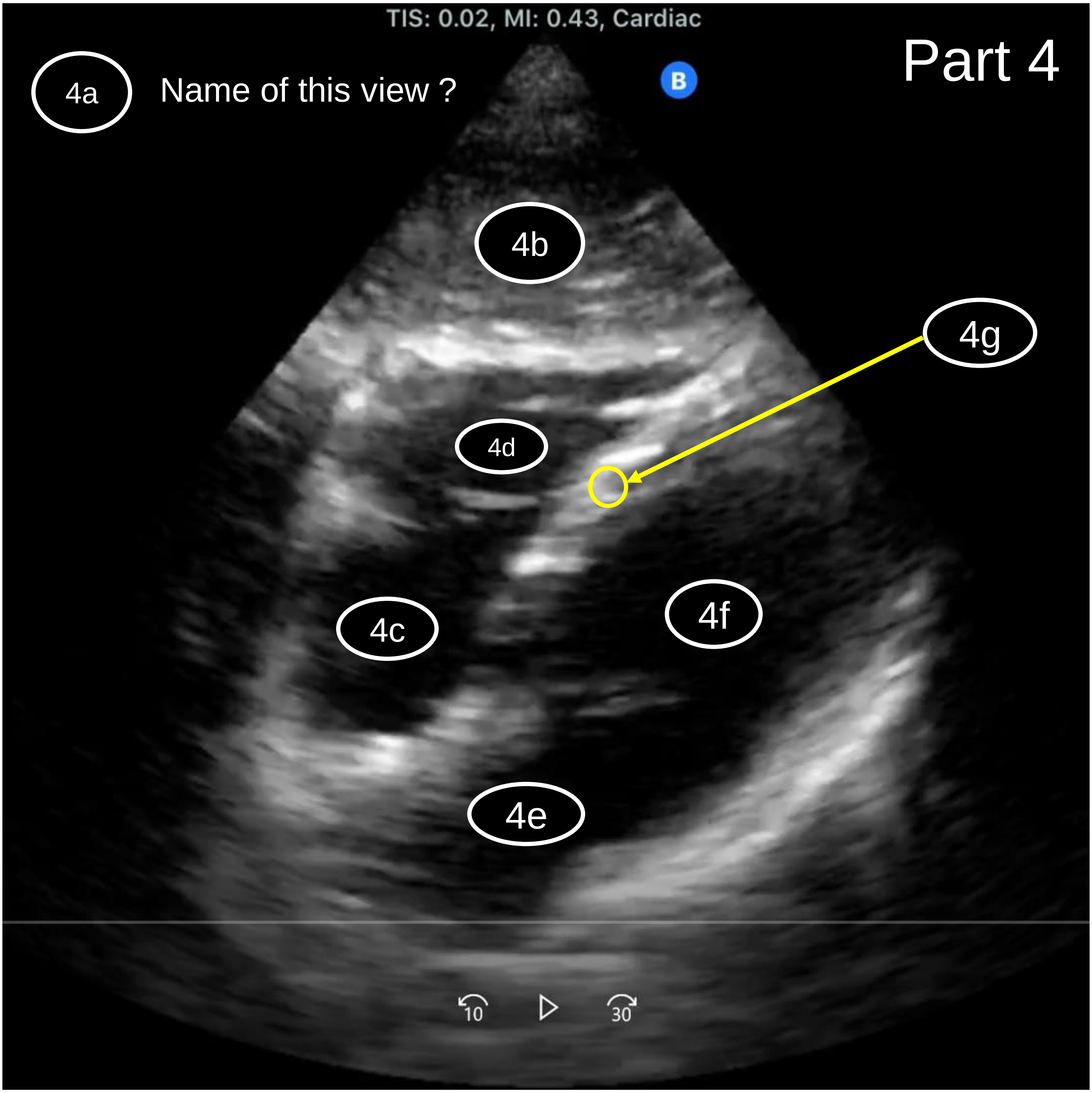

Question 4-1. What is the name of view **4a** ?
Question 4-2. What is **4b** ?
Question 4-3. What is **4c** ?
Question 4-4. What is **4d** ?
Question 4-5. What is **4e** ?
Question 4-6. What is **4f** ?
Question 4-7. What is **4g** ?

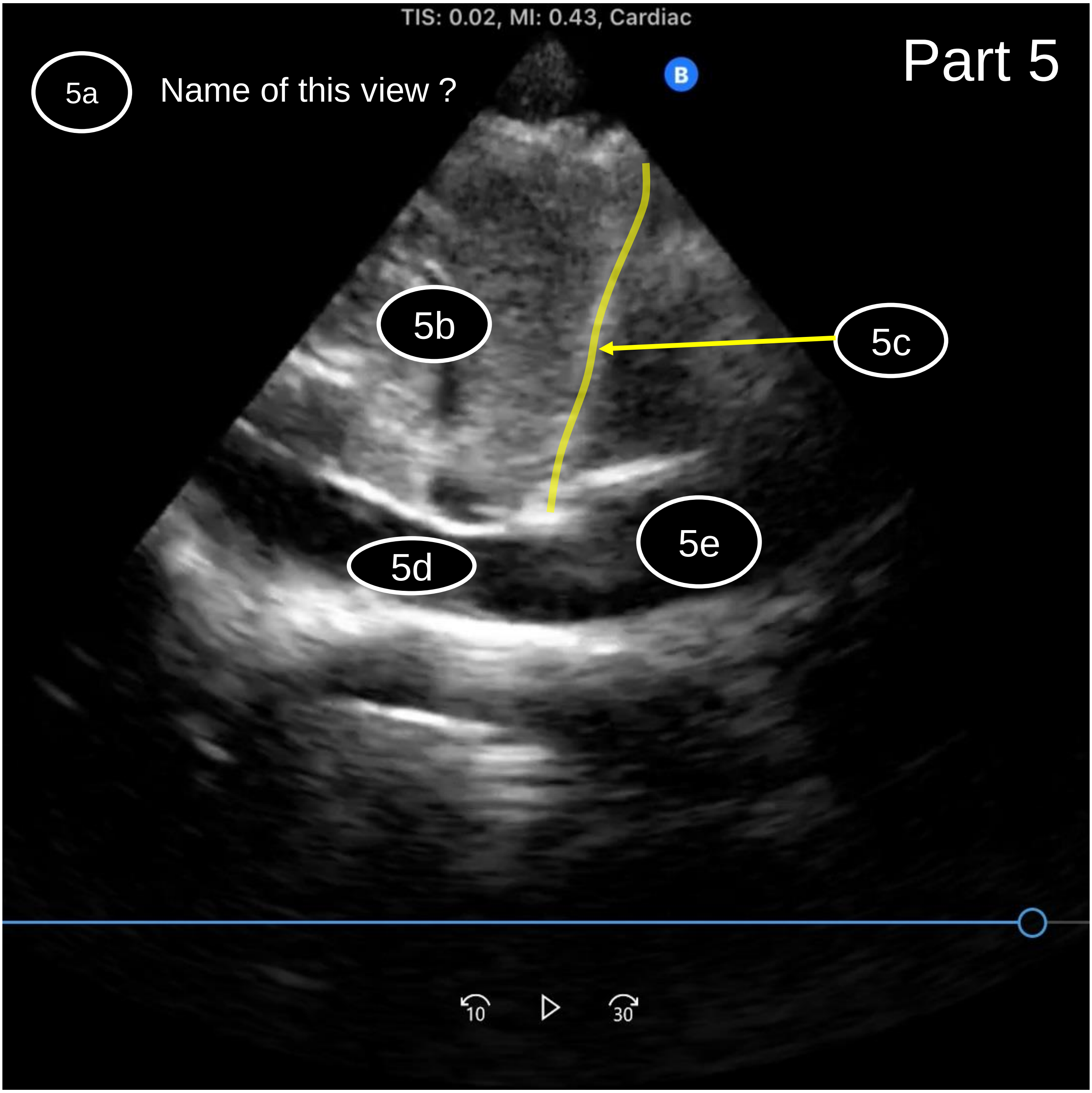

Question 5-1. What is the name of view **5a** ?
Question 5-2. What is **5b** ?
Question 5-3. What is **5c** ?
Question 5-4. What is **5d** ?
Question 5-5. What is **5e** ?

Correct Answers: Part 1 and 2.

Question	Answer
Part 1	
Question 1-1. 1a	31 (Parasternal long-axis view)
Question 1-2. 1b	3 (Right Ventricle)
Question 1-3. 1c	19 (Interventricular Septum)
Question 1-4. 1d	6 (Left Ventricle)
Question 1-5. 1e	7 (Aortic Valve)
Question 1-6. 1f	11 (Ascending Aorta)
Question 1-7. 1g	4 (Left Atrium)
Question 1-8. 1h	8 (Mitral Valve)
Question 1-9. 1i	26 (Pericardium)
Part 2	
Question 2-1. 2a	32 (Parasternal short-axis view)
Question 2-2. 2b	3 (Right Ventricle)
Question 2-3. 2c	6 (Left Ventricle)
Question 2-4. 2d	24 (Postero-medial Papillary Muscle)
Question 2-5. 2e	23 (Antero-lateral Papillary Muscle)
Question 2-6. 2f	19 (Interventricular Septum)
Question 2-7. 2g	20 (Anterior wall of LV)
Question 2-8. 2h	22 (Lateral wall of LV (Antero-lateral))
Question 2-9. 2i	21 (Inferior wall of LV)
Question 2-10. 2j	26 (Pericardium)

LV = Left Ventricle

Correct Answers: Part 3,4, and 5

Question	Answer
Part 3	
Question 3-1. 3a	33 (Apical 4-chamber view)
Question 3-2. 3b	3 (Right Ventricle)
Question 3-3. 3c	9 (Tricuspid Valve)
Question 3-4. 3d	1 (Right Atrium)
Question 3-5. 3e	19 (Interventricular Septum)
Question 3-6. 3f	6 (Left Ventricle)
Question 3-7. 3g	8 (Mitral Valve)
Question 3-8. 3h	18 (Interatrial Septum)
Question 3-9. 3i	4 (Left Atrium)
Part 4	
Question 4-1. 4a	38 (Subcostal 4-chamber view)
Question 4-2. 4b	27 (Liver)
Question 4-3. 4c	1 (Right Atrium)
Question 4-4. 4d	3 (Right Ventricle)
Question 4-5. 4e	4 (Left Atrium)
Question 4-6. 4f	6 (Left Ventricle)
Question 4-7. 4g	19 (Interventricular Septum)
Part 5	
Question 5-1. 5a	39 (Subcostal IVC view)
Question 5-2. 5b	27 (Liver)
Question 5-3. 5c	25 (Diaphragm)
Question 5-4. 5d	17 (IVC)
Question 5-5. 5e	1 (Right Atrium)

IVC = Inferior Vena Cava.
