# Supplementary material for: Pre-clinical medical student cardiac point-of-care ultrasound curriculum based on the American Society of Echocardiography recommendations: a pilot and feasibility study
Source: Pilot Feasibility Stud. 2021 Sep 14;7:175. doi: 10.1186/s40814-021-00910-3 (PMC8438804; doi:10.1186/s40814-021-00910-3)
Supplement: Supplementary file 4 [file 40814_2021_910_MOESM2_ESM.docx]

Additional File 2: Original content of cardiac POCUS lecture

	Item	Content
Part 1	Basic 5 cardiac POCUS views	Introduction to the 5 cardiac POCUS views (PLAX, PSAX, A4C, S4C, and SIVC) with the ASE online module slides and the 5 POCUS views figures from the WINFOCUS recommendations*.
Part 2	Acoustic windows	Utilization of 3 acoustic windows (left parasternal, apical, and subcostal windows) to obtain the 5 cardiac POCUS views.
Part 3	5-view video instructions	Instructions with a pre-recorded video demonstrating how to obtain the 5 views. In the video, the instructor demonstrates the 5 views by manipulating the probe on his own chest wall and demonstrating correctly obtained views. The instructor provides feedback for students' questions about probe manipulation and image acquisition.

POCUS , point-of-care ultrasound; *PLAX* , parasternal long-axis view; *PSAX* , papillary muscle level of parasternal short-axis view; *A4C* , apical 4-chamber view; *S4C* , subcostal 4-chamber view; *SIVC* , subcostal inferior vena cava view; *ASE* , American Society of Echocardiography; *WINFOCUS* , World Interactive Network Focused on Critical UltraSound.

*Via G, et al. 2014. International evidence-based recommendations for focused cardiac ultrasound. J Am Soc Echocardiogr. 27:683.e1-683.e33.
