# Supplementary material for: Pre-clinical medical student cardiac point-of-care ultrasound curriculum based on the American Society of Echocardiography recommendations: a pilot and feasibility study
Source: Pilot Feasibility Stud. 2021 Sep 14;7:175. doi: 10.1186/s40814-021-00910-3 (PMC8438804; doi:10.1186/s40814-021-00910-3)
Supplement: Supplementary file 5 [file 40814_2021_910_MOESM3_ESM.docx]

Additional File 3 : Main instruction points for 5 cardiac POCUS views image acquisition

Cardiac POCUS view	Probe manipulation instructions
Parasternal long-axis view	Place the probe on the 3rd or 4th left intercostal space immediately next to the sternum.<br>And then, rotate the probe so that probe marker points to the patient's right shoulder.
Parasternal short-axis view<br>(Papillary muscle level)	Start from a clear image of parasternal long-axis view and rotate the probe 90 degrees clockwise so that the probe marker points to the patient's left shoulder.<br>And then, tilt the probe downward until both papillary muscles are visualized clearly.
Apical 4-chamber view	Place the probe on the 5th or 6th left intercostal space under the patient's left nipple.<br>And then, tilt up the probe toward the patient's right shoulder until all 4 chambers and the true longitudinal LV cavity are visualized clearly.
Subcostal 4-chamber view	Place the probe immediately below the xiphoid process and point the probe marker to 3 o'clock direction.<br>And then, tilt up the probe toward the patient's left shoulder until all 4 chambers are visualized clearly.
Subcostal IVC view	Start from a subcostal 4-chamber view.<br>And then, rotate the probe 90 degrees counterclockwise so that the probe marker points toward the patient's head.

POCUS , point-of-care ultrasound; *LV* , left ventricle; *IVC* , inferior vena cava.
