# Supplementary material for: Pre-clinical medical student cardiac point-of-care ultrasound curriculum based on the American Society of Echocardiography recommendations: a pilot and feasibility study
Source: Pilot Feasibility Stud. 2021 Sep 14;7:175. doi: 10.1186/s40814-021-00910-3 (PMC8438804; doi:10.1186/s40814-021-00910-3)
Supplement: Supplementary file 6 [file 40814_2021_910_MOESM6_ESM.docx]

Additional File 6: Modified content of cardiac POCUS lecture

	Item	Content	Time
Part 1	Basic 5 cardiac POCUS views	Introduction to the 5 cardiac POCUS views (PLAX, PSAX, A4C, S4C, and SIVC) with the ASE online module slides and the 5 POCUS views figures from the WINFOCUS recommendations*.	2 minutes
Part 2	Acoustic windows	Utilization of 3 acoustic windows (left parasternal, apical, and subcostal windows) to obtain the 5 cardiac POCUS views.	2 minutes
Part 3	Cardiac ultrasound beam shape	Explanation of triangular-shaped cardiac ultrasound beam. The Instructor uses a folding fan and a model heart to illustrate how and why a triangular-shaped beam is emitted from the probe in an easy-to-understand way.	2 minutes
Part 4	Probe orientation marker	Instruction on how to utilize the probe orientation marker and ultrasound display orientation marker for image acquisition. Students place a HHU probe on a model heart and instructor asks questions about the expected images.	2 minutes
Part 5	Probe manipulation	Demonstration of 4 probe manipulations (sliding, rotation, angling, and tilting) on a model heart, showing how the ultrasound beam is moved when applying each manipulation using a folding fan as emitted ultrasound beam. The instructor emphasizes the importance of applying just one manipulation at a time to adjust the image.	2 minutes
Part 6	5-view anatomy	The question-and-answer review of 40 anatomical structures seen in the 5 views using the pre-training knowledge test questions. The instructor gives feedback for incorrect answers.	10 minutes
Part 7	5-view video instructions	Instructions with a pre-recorded video demonstrating how to obtain the 5 views. In the video, the instructor demonstrates the 5 views by manipulating the probe on his own chest wall and demonstrating correctly obtained views. The instructor provides feedback for students' questions about probe manipulation and image acquisition.	10 minutes

POCUS , point-of-care ultrasound; *PLAX*, parasternal long-axis view; *PSAX*, papillary muscle level of parasternal short-axis view; *A4C*, apical 4 chamber view; *S4C*, subcostal 4-chamber view; *SIVC*, subcostal inferior vena cava view; *ASE*, American Society of Echocardiography; *WINFOCUS*, World Interactive Network Focused on Critical UltraSound; *HHU*, hand-held ultrasound.

*Via G, et al. 2014. International evidence-based recommendations for focused cardiac ultrasound. J Am Soc Echocardiogr. 27:683.e1-683.e33.
